# CXCR4/CXCL12 in Non-Small-Cell Lung Cancer Metastasis to the Brain

**DOI:** 10.3390/ijms14011713

**Published:** 2013-01-15

**Authors:** Sebastiano Cavallaro

**Affiliations:** Functional Genomics Center, Institute of Neurological Sciences, Italian National Research Council, Via Paolo Gaifami, 18, Catania 95125, Italy; E-Mail: sebastiano.cavallaro@cnr.it

**Keywords:** brain metastases, chemokines, CXCL12, CXCR4, lung cancer, metastasis

## Abstract

Lung cancer represents the leading cause of cancer-related mortality throughout the world. Patients die of local progression, disseminated disease, or both. At least one third of the people with lung cancer develop brain metastases at some point during their disease, even often before the diagnosis of lung cancer is made. The high rate of brain metastasis makes lung cancer the most common type of tumor to spread to the brain. It is critical to understand the biologic basis of brain metastases to develop novel diagnostic and therapeutic approaches. This review will focus on the emerging data supporting the involvement of the chemokine CXCL12 and its receptor CXCR4 in the brain metastatic evolution of non-small-cell lung cancer (NSCLC) and the pharmacological tools that may be used to interfere with this signaling axis.

## 1. Introduction

Chemokines (chemotactic cytokines) are small, mostly secreted proteins that are implicated in many biological processes. Although the chief role for chemokines is differential regulation of leukocyte trafficking in hematopoiesis and in innate and adaptive immunity, they exert several other functions, such as antimicrobial activity, embryogenesis, angiogenic or angiostatic activity, hematopoiesis, apoptosis or mitogenic activity, and tumor-promoting or tumor-inhibiting activity [1–4]. They comprise a family of small (~70–80 amino acids in length), mostly secreted proteins, which can be classified into CXC, CC, C or CX3C subfamilies based on the arrangement of the two cysteine residues near the *N*-terminus [5]. Most chemokines belong to the CC and CXC classes, whereas there are only two known C chemokines, and one known CX3C chemokine. Chemokines act by binding to 7-transmembrane domain, G protein-coupled receptors. To date, 18 human chemokine receptors [6–9] and over 50 distinct chemokines [5] have been described. Some of these receptors are highly selective for one endogenous chemokine ligand (monogamous receptors), whereas others are highly promiscuous and may be activated by more than one chemokine.

An important member of the CXC chemokine subfamily, CXCL12 has been widely explored in cancer [10–13]. The cDNA encoding CXCL12 was originally cloned from a murine bone marrow stroma cell line and its gene was originally named stromal cell-derived factor-1 (SDF1) [14]. Few years later, two groups identified its receptor, an orphan GPCR called LESTR/fusin [15,16]. This receptor was also the first identified receptor for human immunodeficiency virus (HIV-1) infection of CD4^+^ lymphocytes [17] and CXCL12 was able to inhibit infection by T-tropic HIV-1 of HeLa-CD4 cells [15,16]. Based on its ability to bind and respond to CXCL12, the designation CXCR4 was later used to replace LESTR/fusion.

**Figure 1:**
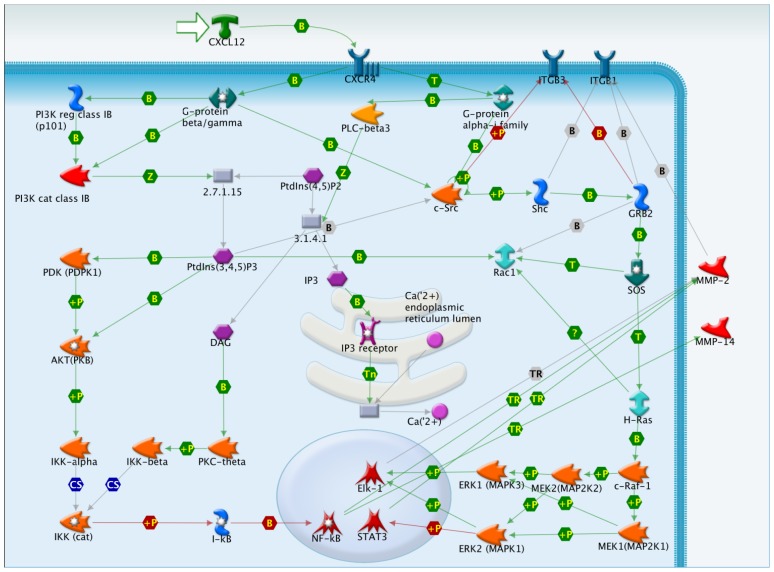
Intracellular CXCR4/CXCL12 signaling. Association of CXCL12 with CXCR4, a G-protein couple receptor, is known to activate multiple signaling pathways. Interaction, relation and color labels: B, binding; +P, phosphorylation; TR, transcription regulation, Z, catalysis; CS, complex subunit; green link, positive effect; red link, negative effect; grey link, unknown effect.

Association of CXCL12 with CXCR4 is known to activate multiple signaling pathways (Figure 1) [18–25]. Activation of CXCR4 by CXCL12 promotes interaction between the receptor and the trimeric G-protein alpha (i), beta/gamma. This causes the exchange of GDP for GTP bound to G protein alpha subunits and the dissociation of the beta/gamma heterodimers. The G-protein beta/gamma heterodimers activate PI3K gamma, recruiting non catalytic p101 subunit and directly stimulating catalytic p110 gamma subunits. PI3K converts phosphatidylinositol 4,5-biphosphate to phosphatidylinositol 3,4,5-triphosphate [PI(3,4,5)P3]. PI(3,4,5)P3 is a second messenger that directly binds to PtdIns (3,4,5)P3-dependent protein kinase-1 (PDK1) and protein kinase B (AKT). PDK phosphorylates AKT, AKT in turn, activates Inhibitor of nuclear factor kappa-B kinase (IKK), and IKK phosphorylates the I-kappa-B (I-κB) proteins, making them available for destruction via the ubiquitination pathway, thereby allowing activation of the NF-kappa-B (NF-κB) complex. Activation of phospholipase C (PLC) results in hydrolysis of phosphatidylinositol (4,5)-bisphosphate (PtdIns(4,5)P2) and generation of the second messengers 1,2-diacylglycerol (DAG) and inositol (1,4,5)-trisphosphate (IP3). DAG is a physiological activator of Protein kinase C (PKC) whereas IP3 binds to a specific receptor present on the endoplasmic reticulum, resulting in the release of intracellular stored Ca(2+). PKC can signal through IKK. G-alpha-protein directly stimulates kinase activity of the Src family kinase tyrosine-protein kinase c-Src, binds to the catalytic domain and changes the conformation of c-Src. In turn, c-Src activates H-Ras-c-Raf-1-MEK1/2-ERK1/2 pathway through phosphorylation of adaptor protein Shc and recruitment of adaptor protein GRB2 and positive regulator of RAS guanine nucleotide exchange protein SOS, leading to the increased transactivation ability of transcription factor Elk1 and the repressed transactivation ability of transcription factor STAT3 which both are phosphorylated by ERK2. Although the exact CXCR4/CXCL12 signaling pathways implicated in lung cancer metastasis are almost unknown, CXCR4 regulates migration of lung cells through activation of Rac1 and matrix metalloproteinases (MMP-2 and MMP-14) [19], and through the combined action of ERK, IKK, NF-kappaB and integrins (ITGB1 and ITGB3) [22].

In addition to their pleiotropic effects, activation of CXCR4 by its ligand CXCL12 has been shown to play an important role in growth and metastasis of various tumors [10,11,26–30]. This review will focus on the emerging data supporting the putative involvement of the CXCR4/CXCL12 signaling axis in non-small-cell lung cancer (NSCLC) metastasis to the brain. These insights are providing new opportunities to improve current therapies that address lung cancer spread.

## 2. NSCLC Metastasis to the Brain

NSCLC accounts for 85% of all cases of lung cancers and adenocarcinoma is the most common histologic subtype [31–33]. Despite advances over the last decade in diagnostic, staging and surgical techniques, as well as new pharmacological and radiotherapy protocols, lung cancer represents the leading cause of cancer-related mortality in both men and women throughout the world. Patients die of local progression, disseminated disease, or both. Following its primary development, NSCLC can progress along three different courses, which often occur together: local invasion of adjacent structures (mediastinum and the chest wall), lymphatic spread to regional lymph nodes, and distant hematogenous metastasis. The most common sites of metastasis are the bones, liver, adrenal glands, pericardium, brain, and spinal cord [34]. Synchronous or metachronous brain metastasis occur in approximately 33% of NSCLC patients [35]. Lymph node involvement at diagnosis confers a higher risk of CNS recurrence [36,37]. In locally advanced (LAD)-NSCLC, brain metastasis are reported in 17% to 38% of patients, with a median time-to-brain relapse in the range of 7.5–9.3 months [38–41]. In this setting, brain metastasis are mostly observed within two years from initial diagnosis [38,42] being the first site of failure in 29% of all recurrences, and the exclusive site of recurrence in 72% of these cases (21% of all recurrences). Among brain recurrences, 36% of them are single brain metastases, whereas multiple brain metastases occur in the rest of the cases [43]. Overall, solitary metastases are reported in approximately 30% of NSCLC [35].

In LAD-NSCLC, age and other clinical features (complete *versus* incomplete resection, non-squamous *versus* squamous histology, size of the primary tumor, and adjuvant chemotherapy *versus* none) are clinical prognostic indicators of brain metastasis occurrence [40,43,44]. The number of mediastinal lymph node regions involved and the overall number of mediastinal metastases (less than 4, 4–6, and more than 6) are also significantly associated with the frequency of brain metastasis in LAD-NSCLC. Based on clinical data, a mathematical model to predict brain metastasis risk was proposed with the aim of aiding in selection of patients with LAD-NSCLC for prophylactic cranial irradiation in clinical trials [40].

Regarding chemotherapy, brain has been reported as a frequent site of disease recurrence in patients with NSCLC after multimodality therapy and an initial response to the tyrosine-kinase inhibitor gefitinib [45,46]. In the latter study, brain was the first site of disease recurrence in 33% and the sole site of disease progression in 57% of the cases. Several reasons may explain this finding: the resistance of tumor metastatic clones, incomplete CNS penetrance of gefitinib, longer survival of patients treated with gefitinib, and possible difference in tumor biological characteristics, such as the status of the EGFR receptor [43,47]. The high rate of isolated brain metastasis in LAD-NSCLC patients after multimodality treatment, has suggested some authors a renewed interest in prophylactic cranial irradiation [41,48], as well as new strategies of follow-up aimed to increase the chances of effective and timely treatment [42,49]. In a German multicenter randomized trial [50], the addition of prophylactic cranial irradiation within a trimodality treatment protocol (chemotherapy, chemoradiotherapy, surgery) for patients with operable stage IIIA NSCLC was effective in preventing brain metastasis with no significant neurological/cognitive related late effects. The use of routine scans of the brain in follow-up examinations and prophylactic chemotherapy in patients at high-risk of brain metastasis, therefore, are potentially useful options but still need to be validated in clinical controlled trials.

## 3. CXCR4 and CXCR12 in NSCLC Metastasis to the Brain

Chemokines and chemokine receptors could be important in explaining the different propensity to brain metastatization among different NSCLCs. Chemokines selectively regulate the recruitment and trafficking of leukocyte subsets to inflammatory sites through chemoattraction and by activating leukocyte integrins to bind their adhesion receptors on endothelial cells [51]. The mechanisms involved in leukocyte trafficking may also be used by tumor cells, and a chemokine gradient (migration is towards increasing chemokine concentration) may be established between the chemokine receptor of a cancer cell and the respective ligand expressed at sites of tumor spread. Indeed, different chemokines and their respective receptors have been implicated in the development of primary tumor and metastases, providing biological support of the “seed and soil” theory [52–54]. The next paragraphs will review the potential role of CXCR4 and CXCR12 in NSCLC metastasis to the brain.

CXCR4 is expressed by a majority of tumors, including those of epithelial, mesenchymal and hematopoietic origin, and it appears to be a ubiquitous receptor [55]. Based on the well-characterized roles of CXCL12 and CXCR4 in chemotaxis and the similarities between chemotactic cell migration and cancer cell movement to distant sites, this receptor-ligand pair has been hypothesized to play a role in cancer pathogenesis and metastasis. The CXCR4-CXCL12 interaction and downstream signaling has been shown to promote growth/survival of tumor cells and allow them to grow in distant and less favorable sites [24,56–59]. CXCR4 expression has been identified as a predictive factor of worse outcome in some metastatic tumors and in malignant gliomas [60]. CXCL12/CXCR4 axis is supposed to be crucial in brain metastasis formation from breast cancer [12].

In lung cancer, in particular, several studies have demonstrated a correlation between CXCR4 expression and clinical outcomes, with increased expression in tumor tissue over normal lung tissue, and increased expression in tumors of patients with metastatic disease *versus* those without clinical metastasis [61–69]. In a recent study, we have investigated the expression of CXCR4, together with those of its ligand CXCL12, in primary NSCLC specimens of patients with and without brain metastasis, using a quantitative double-labeling immunofluorescence analysis [70]. We matched a M0 NSCLC group with a M1 NSCLC group, using clinical and pathological characteristics (gender, age, histology, T stage, and N stage) as consistent as possible. The results obtained showed that CXCL12 and CXCR4 immunoreactivities in M1 NSCLC samples were significantly higher than that in paired M0 NSCLC. Altogether, these findings support a role of CXCL12 and CXCR4 in the process of NSCLC metastatic spread to brain.

### 3.1. CXCR4 Signaling in NSCLC Metastasis

The metastatic potential of NSCLC is dependent on several orchestrated events, such as active locomotion, extracellular matrix degradation, and adhesion to vascular endothelial cells. Although there has been limited research on the signal transduction pathways mediated by CXCR4 in lung cancer cells (Figure 1), some of the mechanisms involved in NSCLC metastasis are beginning to be elucidated. CXCL12 was shown to increase the migration of lung cancer cells through the CXCR4-mediated activation of ERK, which in turn activates IKKa/b and NF-κB, resulting in the activations of integrins (ITGB1 and ITGB3) [22]. In addition to integrin activation and signaling, CXCR4 also stimulates the production of matrix metalloproteases in lung cells. CXCR4, for example, has been recently shown to regulate migration of lung cells also through activation of Rac1 and matrix metalloproteinases (MMP-2 and MMP-14) [19]. Additional studies are still required to identify the signaling pathways by which CXCL12 and CXCR4 may regulate NSCLC metastasis. Since their actions influence gene expression, microarray analysis could be employed as it has been done to investigate CXCL12 induced signaling in T cells [71], breast cancer [72] or glioma cells [73].

### 3.2. CXCL12 and CXCR4 Expression in NSCLC

When CXCR4 is blocked by the antagonist AMD-3100, or knocked down by short hairpin RNA, cell migration is significantly inhibited [19]. If CXCR4 truly mediates metastasis, when lung cancer cells enter the blood or lymphatic systems, they would preferentially migrate and adhere to areas with high expression of CXCL12. Previous *in vitro* studies have shown that NSCLC cell lines express high levels of CXCR4 and that CXCL12-activated CXCR4 promotes migration and invasion of these cell lines [74].

Different factors can also influence CXCL12 and CXCR4 expression in lung cancer. Hypoxia has been shown to promote CXCR4 expression in NSCLC. Overexpression of the epidermal growth factor receptor (EGFR) is associated with the majority of NSCLC and has been implicated in the process of malignant transformation by promoting cell proliferation, cell survival, and motility. Activation of EGFR by the Epidermal Growth Factor (EGF), especially under hypoxic conditions, increases CXCR4 expression and the migratory capacity of NSCLC cells [75]. This EGFR-mediated increase of CXCR4 appears to be mediated by phosphatidylinositol 3-kinase/PTEN/AKT/mTOR signal transduction pathway, and by the activation of hypoxia inducible factor (HIF) 1α, a transcription factor that allows tumor to prosper under conditions of low oxygen tension.

CXCR4 downregulation by an antisense nucleotide fragment or a neutralizing antibody significantly decreases migration, invasion, and adhesion of NSCLC cell line cells [67]. *In vivo* studies using a mouse model of heterotopic or orthotopic xenoengraftment of human NSCLC cells show that preferential sites of lung cancer metastases have significantly higher levels of CXCL12 protein expression than the primary tumor or plasma levels, suggesting that a chemotactic gradient may be established between the site of the primary tumor and metastatic sites [74]. Neutralization of CXCL12 by an anti-CXCL12 or anti-CXCR4 monoclonal antibody resulted in a significant decrease of NSCLC metastases to several organs including the adrenal glands, liver, lung, brain, and bone marrow [74,75]. Similar results were found in a retrospective clinical study showing a correlation between primary tumor CXCR4 expression and clinical outcome in NSCLC patients [67].

In line with the above studies, CXCL12 and CXCR4 may be used as markers of risk-prediction for metastasis in the initial staging of NSCLC patients. In order to assess this hypothesis, we performed Receiver Operating Characteristics (ROC) analysis in order to define optimal cut-off values for CXCL12 and CXCR4 immunoreactivities that could discriminate between NSCLC patients without and with brain metastasis [70]. ROC curves showed a good diagnostic accuracy and adequate predictive power for both CXCL12 and CXCR4, supporting their potential use as prognostic markers. However, since our analysis utilized a retrospective cohort of NSCLC patients and a small sample size, the results obtained should be confirmed in a larger study.

Another issue that remains to be investigated regards the role of CXCL12 and CXCR4 in the evolution of NSCLC metastatic traits and their affinity for brain. To this end, it would be useful to characterize the expression of CXCL12 and CXCR4 in NSCLC brain metastasis and correlate the sites of CXCL12 expression in the brain with the site of metastasis formation. In regard to the latter, it is important to point out that both CXCL12 and CXCR4 are constitutively expressed in the brain by neurons and glial cells, and have been involved in several brain processes, such as development, cell migration, neuronal survival, and neurotransmission [76]. Furthermore, the expression of CXCL12 and CXCR4 can be altered during pathological conditions [77–79]. Moving from the primary tumor to the site of metastasis may not be easy, since candidates for surgery removal of brain metastasis constitute a very small percentage of NSCLC patients.

Although further studies are still needed to better evaluate the role of CXCL12 and CXCR4 in the process of NSCLC metastatic spread to brain, their use as predictive markers of metastasis in brain (with or without extracranic sites) may be clinically relevant for the invalidating consequences on patient’s autonomy and quality of life, and the opportunity to implement radiological surveillance.

## 4. Antagonist of CXCR4-Mediated Brain Metastasis

In consideration of the role of chemokines and their receptors in tumor growth and metastasis, a number of therapeutic agents that specifically target chemokine receptors have been used for cancer therapy. Drugs (small molecule or peptide inhibitors) targeting chemokine receptors or monoclonal antibodies blocking their ligands were shown, both *in vitro* and *in vivo*, to inhibit tumor cell growth and prevent metastases [80,81].

CXCR4 antagonists include low-molecular-weight molecules, such as AMD3100 and MSX-122, and peptides, such as ALX40-4C or the polyphemusin analogues (TN14003/BKT140), T22, and CTCE-9908 [82].

Although several studies have assessed the antimetastatic effects of CXCR4 inhibitors in different cancer types [83–98], only few have investigated their effects in lung cancer. TF14016, a small peptidic inhibitor of CXCL12 receptor CXCR4, has been recently shown to suppress metastases of small-cell lung cancer cells in mice [99]. Treatment with AMD3100 (also known as Plerixafor and Mozobil), a bicyclam currently used in the mobilization of hematopoietic stem cells from the bone marrow [100], or TN14003/BKT140 were shown to disrupt CXCR4-mediated tumor cell adhesion to stromal cells and sensitize lung cancer cells to cytotoxic drugs [101,102]. In addition to their ability to sensitize NSCLC cells to conventional anticancer therapies, CXCR4 antagonists are known to directly influence the NSCLC metastatic spread to brain. An *in vitro* study showed that blockade of the CXCR4/CXCL12 axis by transfection with a CXCR4 antisense nucleotide fragment or by a CXCR4 neutralizing antibody significantly decreases migration, invasion, and adhesion of NSCLC cell line cells [67]. *In vivo*, depletion of CXCL12 by the administration of neutralizing anti-CXCL12 antibodies in immunodeficient mice expressing human NSCLC significantly impairs metastases to the adrenal glands, bone marrow, liver, and brain [103]. More recently, BKT140, a highly selective inverse agonist of CXCR4, was shown to reduce the colony-forming capacity of NSCLC cell lines *in vitro*, and the growth of NSCLC cell line xenografts *in vivo* [104]. Although these findings are very encouraging, additional studies are still necessary to test the safety and efficacy of CXCR4 inhibitors against lung cancer brain metastasis.

## 5. Conclusions

There is emerging evidence to support a role for the CXCR4/CXCL12 signaling axis in the brain metastatic evolution of NSCLC. The field is now ready to move from preclinical research into clinical trials where the role of CXCR4/CXCL12 axis, together with the safety and efficacy of CXCR4 inhibitors in NSCLC, should be fully assessed.
